# Subcutaneous Dextrose Injection Above Muscle Insertions of 4/5 Muscles Restores 5/5 Power in Patients With Distant Cerebrovascular Accidents, Multiple Sclerosis, or Radiculopathy: Five Case Reports

**DOI:** 10.7759/cureus.92073

**Published:** 2025-09-11

**Authors:** Kim C Meyers, Brian McDonagh, Aeneas Janze, Ian M Thornell, K. Dean Reeves

**Affiliations:** 1 Internal Medicine, Endeavor Health, Evanston, USA; 2 Vein Therapy, Independent Researcher, Glenview, USA; 3 Physical Medicine and Rehabilitation, Walter Reed National Military Medical Center, Bethesda, USA; 4 Internal Medicine-Physiology Subsection, University of Iowa Carver College of Medicine, Iowa City, USA; 5 Rehabilitation Medicine, Independent Researcher, Kansas City, USA

**Keywords:** dextrose 5%, ischemic cva, lumbar disc disease, motor deficit, multiple sclerosis and other demyelinating disorders, neuroplasticity enhancement, neuroprolotherapy, perineural injection therapy, stroke, weakness in limbs

## Abstract

In 2011, a patient with dense distal sensory loss from diabetic neuropathy and 4/5 ankle dorsiflexion power was treated with subcutaneous injection of D5W in the region of the interdigital nerves. Upon injection completion and coming to stand, the patient remarked, "I can feel the floor and move my toes more." Great toe and ankle dorsiflexion could not be overcome by manual testing after injection. Further empirical observations by three authors trialing the method (KCM, BM, KDR) have suggested frequent gains from 4/5 strength (active movement against gravity and resistance) to 5/5 strength (normal power) within 3 minutes after subcutaneous injection of related muscle insertions in patients with motor weakness from distant upper or lower motor neuron conditions. We have not observed a similar improvement with injection of normal saline or sterile water over muscle insertions, or with D5W injection subcutaneously over muscle origins or bellies. This treatment will be referred to as Dextrose Muscle Insertion Therapy (DMIT).

Five case reports are presented, with videos showing within-session restoration of 5/5 strength in an ischemic cerebrovascular accident (CVA) patient with chronic ankle dorsiflexion weakness 30 years post event, a relapsing-remitting MS patient with deltoid weakness one year following an exacerbation of MS, a diabetic patient with deltoid weakness six years post cervical radiculopathy, a patient with post-injury disuse-related wrist extension weakness 14 years post injury, and a patient treated for left upper extremity weakness at time of an apparent plateau seven weeks post ischemic CVA. These videos are not time-stamped, nor are these observations controlled. The specificity of the injection site, rapid response, single treatment sufficiency, magnitude of response, and observation of similar responses by multiple physicians argue against a placebo response.

This discovery, if confirmed, may challenge the long-held belief that motor recovery or muscle recruitment plateaus permanently after injury and prompt additional research on the neurophysiology of muscle recruitment limitations after distant events. Confirmatory case series with time-stamped video confirmation and controlled clinical trials should follow to confirm these observations, their frequency, responsive diagnoses, and optimum time periods for treatment following the onset of weakness.

## Introduction

Neurologic injuries from cerebrovascular accident (CVA; ischemic or hemorrhagic), multiple sclerosis (MS), spinal cord trauma, or nerve root compression are commonly followed by a component of permanent motor weakness. Even after extensive physical and occupational therapy and the passage of time, many patients will be left with 4/5 muscle weakness [1] on the 0-5 Medical Research Council (MRC) [2] scale for muscle power.

Perineural injection therapy (PIT) using dextrose 5% in water (D5W), in which D5W was delivered subcutaneously over painful peripheral nerve pathways, was introduced by Dr. John Lyftogt in 2006 as a treatment for chronic pain [3]. The therapeutic efficacy of subcutaneous dextrose injection was first evidenced in a controlled clinical trial in 2009 [4].

In 2011, a patient with dense distal sensory loss from diabetic neuropathy and 4/5 ankle dorsiflexion power was treated using subcutaneous injection of D5W (perineural injection therapy (PIT)) [5] by Dr. Kim Meyers. The patient indicated feeling stronger in her feet, and her great toe and ankle dorsiflexion tested as 5/5 in power post-treatment. This observation was followed by years of trial-and-error treatment with D5W, focusing on patients with 4/5 power in a variety of muscles and across both upper and lower motor neuron conditions. Ultimately, it was discovered that the injection had to be near the muscle's distal tendon insertion point to get the gain in muscle strength. This suggested a common mechanism of action.

Similar empirical observations have been made by three of the coauthors of this paper (KCM, BM, and KDR). These include that subcutaneous injection of D5W has to be near the insertion point of the tendon to have a positive effect, effects occur within 3 minutes, favorable effects appear to be durable, and repeat treatment does not appear necessary. The range of time between onset and treatment of patients with muscle weakness of 3/5 or 4/5 has been from a little as six weeks to greater than 30 years. Injection of D5W over muscular origins or muscle bellies has not empirically produced similar responses, and neither normal saline nor sterile water injections have resulted in a similar response with injection over muscular insertions.

The frequency of favorable responses has not been formally measured and is pending results from consecutive patient or randomized controlled trials in the design stage at the time of this writing.

This treatment will be referred to as dextrose muscle insertion therapy (DMIT).

## Case presentation

The venue for these cases was an outpatient clinic. Although we consider these cases to be representative, these were not consecutive and were gathered from 2011 through 2024.

Table 1 lists age, diagnosis, duration of weakness in months, muscle action tested, manual muscle testing result pre-injection, time of testing post-injection, and manual muscle testing result post-injection. Table 1 suggests a wide variation in age, diagnoses, and duration of weakness. Testing post-treatment was prompt, as short as 1 minute but not longer than 3 minutes but was not precisely recorded. Four over five power covers a large range with respect to muscle power, representing the ability to move against gravity at full range and against some resistance [2]. Despite variable baseline power in the 4/5 range, power testing comparing the same muscle action on the affected and non-affected sides revealed empirical demonstration of symmetrical power post-treatment.

**Table 1:** Cases presented ^a ^Testing dexterity, not motor power, of fingers MMT: manual muscle testing

Case No	Age	Diagnosis	Weakness duration in months	Action	MMT Pre	Time for post MMT	MMT Post
1	67	R basal ganglia CVA	360	Ankle and Toe Dorsiflexion	4/5	≤3	5/5
2	57	Multiple sclerosis	12	Shoulder elevation	4/5	≤3	5/5
3	78	Cervical radiculopathy	31	Shoulder elevation	4/5	≤3	5/5
4	59	Disuse weakness	168	Wrist extension	4/5	≤3	5/5
5	83	R frontal CVA	2	Elbow extension	4/5	≤3	5/5
				Elbow flexion	4/5	≤3	5/5
				Wrist extension	4/5	≤3	5/5
				Pronation	4/5	≤3	5/5
				Supination	4/5	≤3	5/5
				Finger dexterity	^a^	≤3	^a^
				Shoulder elevation	4/5	≤3	5/5

Description of technique

The technique is simple. Depending on the size of the muscle insertion space, a 1-, 3-, or 5-mL syringe is filled with D5. The syringe is fitted with a 27- or 30-gauge needle. After standard informed consent and aseptic preparation, the needle is angled at 45 degrees, inserted through the skin, and advanced with a shallow angle toward the subcutaneous area directly superior to the muscle insertion point. The amount of subcutaneous space to inject into makes a difference. For example, 0.5 mL would be a typical amount of D5W for the extensor hallucis longus tendon insertion, while 5 mL would be a typical volume of D5W for the deltoid and quadriceps insertions. In addition, the starting point for digits, such as the extensor hallucis longus, would only be about 1.25 cm proximal to the insertion point, compared to 2.5-4.0 cm for a large muscle.

General observations

The most important predictor of response is baseline strength at the time of treatment. The most reproducible response is in patients with 4/5 baseline strength.

Positive responses, such as those seen in the videos included, have occurred in an estimated 100 or more patients over 14 years of experience, many of whom had multiple muscles with 4/5 weakness treated. Changes in activities of daily living (ADL) performance have paralleled improvement in muscle strength gains. ADL improvements appear to have been empirically significant in those with 3/5 strength as well, such as improvements in lifting a hemiplegic leg into a car or up a stair. Patients with 3/5 baseline strength have not been observed to recover normal power, although systematic follow-up with repeated sessions has not been performed. Clinically significant improvement in those with less than 3/5 strength has not been empirically observed.

Case one: distant ischemic CVA

**Figure 1:**
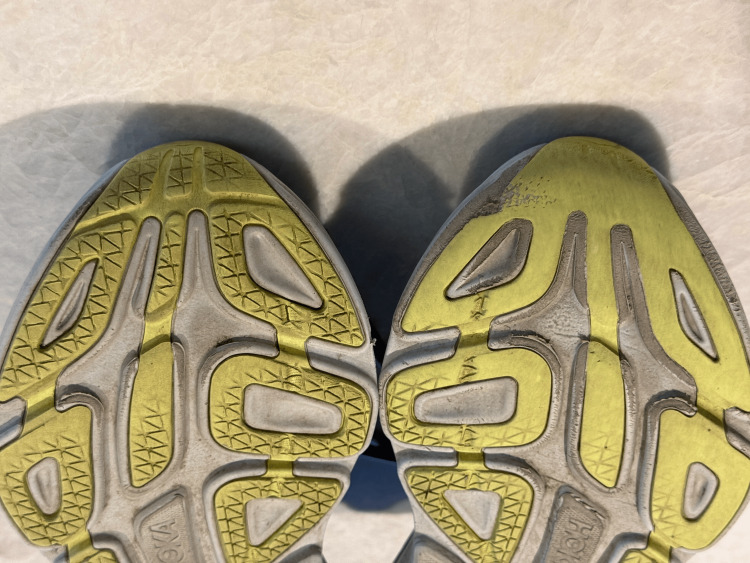
Picture of soles of patients’ shoes The tread pattern is objective evidence of the consequences of her stroke.

This 67-year-old female experienced an ischemic right basal ganglia CVA 30 years prior, at age 37. After one year of physical rehabilitation, she had residual left-sided foot drop due to 4/5 dorsiflexion weakness. The effects on her shoes of a consistent forefoot-prior-to-heel contact (toe-to-heel rather than heel-to-toe) gait pattern with the left foot are demonstrated in Figure 1, which is a still picture taken at the 4-minute and 20-second mark during video follow-up, when her previous shoes were held up for examination.

The treatment received was an injection of 1 mL of D5W targeting insertions of the left hallucis longus and extensor digitorum longus tendons. The point of entry of the needle was approximately 1.25 cm proximal to each tendon insertion. The primary author observed in prior cases that treatment of the tibialis anterior was not required in most cases. As shown in Video 1, the patient’s strength was 5/5 post-treatment of her hallucis longus and extensor digitorum longus insertions; therefore, the anterior tibialis insertion area was not injected.

Video 1 was taken before and after treatment of her left-sided 4/5 dorsiflexion weakness. The first part of Video 1 shows within-session improvement from 4/5 to 5/5 in dorsiflexion strength. Video 1 continues and shows the same patient six weeks later, with her left ankle dorsiflexion strength still 5/5.

**Video 1:** 67-year-old female 30+ years from basal ganglia CVA. 4/5 dorsiflexion treated with subcutaneous D5W above muscle insertion resulted in 5/5 strength. The latter part of the video shows that she continued to have 5/5 dorsiflexion in her left foot six weeks later.

She and her husband confirmed that during pre-treatment, she was catching her foot with near-balance loss several times daily. After treatment and consistently through six weeks post-treatment, catching her left foot had completely stopped.

Both she and her husband describe that she likes to hike, and post-treatment, she walks on level and hilly terrain without tripping. Last contact with the patient at six months after DMIT confirmed that functional improvements were persistent.

Case two: deltoid weakness from multiple sclerosis

This 57-year-old male noted the onset of weakness issues in 2011 but did not seek diagnostic help until 2019, when he developed sudden left-sided weakness. He was diagnosed by a neurologist with relapsing-remitting multiple sclerosis. Flares continued, and he regressed functionally from requiring a cane to needing a walker. Flares decreased following initiation of Ocrelizumab. His last minor MS flare was in 2024, approximately one year prior to being seen in the primary author’s clinic. On the day of his first evaluation on 3/21/25, his left deltoid was treated. Treatment involved needle entry approximately 2.5 cm proximal to the estimated deltoid insertion, advancing to inject 5 mL of D5W subcutaneously above the deltoid insertion. 

In Video 2, left deltoid power improved from 4/5 to 5/5 within minutes. Video 2 continues on to show the durability of DMIT with unabated 5/5 left deltoid strength a full four months after his treatment.

**Video 2:** 57-year-old male with deltoid weakness from multiple sclerosis diagnosed in 2019. Rapid strength improvement with DMIT. Left deltoid strength is still 5/5 two months later.

Case three: deltoid weakness post radiculopathy in a diabetic with demyelinating peripheral neuropathy

This 78-year-old male was diagnosed with type 2 diabetes at age 38 and subsequently was managed with diet and oral medication. In 2009, he developed painless subacute weakness in his left arm. Neurology consultation led to several blood tests to exclude neoplastic syndromes and metabolic neuropathies. A cervical magnetic resonance imaging (MRI) scan in September 2009 showed no clearly concordant findings, with only moderate left foraminal stenosis at C5-6, and an MRI of the left brachial plexus was unremarkable. Electromyographic findings in November 2009 and November 2012 showed a moderate to severe motor and sensory axonal and demyelinating peripheral neuropathy. His strength was stable until March of 2015, at which time he reported difficulty playing the piano with his left hand, and atrophy of the deltoid, triceps, and biceps was noted on examination. His cervical MRI findings showed a left paracentral disc protrusion at C6-7 and moderate left C5-6 foraminal stenosis. Electromyography revealed a left C6 radiculopathy, with both chronic and active components, which did not clearly explain the C5 muscle (deltoid) atrophy. The impression was multifactorial worsening of left upper arm weakness with potential contributions from the underlying sensorimotor axonal and demyelinating polyneuropathy, superimposed upon recent C6 radiculopathy, and potential rotator cuff dysfunction. On June 6, 2015, the subcutaneous region above the left deltoid insertion was injected with 5 ml of D5W, beginning 2.5 cm proximally.

Video 3 demonstrates his 4/5 multifactorial deltoid weakness and shows 5/5 left deltoid strength approximately 3 minutes post DMIT.

**Video 3:** 4/5 deltoid weakness from cervical disc disease and axonal and demyelinating peripheral neuropathy Strength improved from 4/5 to 5/5 minutes after DMIT.

His return of 5/5 deltoid strength was demonstrated again at a seven-week follow-up visit on August 1, 2015. The patient stated he was “reaching for coffee cups on the top shelf better and easier,” and manual reexamination confirmed 5/5 left deltoid power. 

Case four: wrist extension weakness post-fractures without documented neurologic involvement, presumably related to disuse weakness

This 59-year-old male suffered multiple wrist fractures following a soccer injury at age 45. Wrist extension power and wrist range of motion were limited by pain for several months. Pain resolved, but wrist extension weakness persisted and was especially evident if the object's shape required him to use his thumb and all his fingers. In March 2015, he was treated with DMIT with an injection of 0.5 ml of subcutaneous D5W above the insertion of each extensor digitorum along with the insertion points of the extensor carpi radialis and ulnaris. Video 4 was taken before and just minutes after the injection.

**Video 4:** Weakness of wrist extension from disuse Response to DMIT within minutes despite weakness being present for over 13 years

Wrist extensor power improved from 4/5 to 5/5 within the session. In a letter dated May 9, 2025, 10 years following treatment, he indicated having no difficulty extending the left wrist, even when lifting weighted objects. 

Case five: subacute ischemic CVA, at the time of an apparent plateau in improvement

This 83-year-old female awoke on May 26, 2015, with more than left-hand and arm weakness. She was admitted and found to have a small infarct in the right posterior frontal parenchyma on MRI. When she was seen in the internal medicine clinic for follow-up on July 21, 2015, for blood pressure management, she stated that she was going to be discharged soon from outpatient rehabilitation due to a plateau in improvement. In addition to medical management of her hypertension, she consented to a treatment trial with DMIT. Treatment consisted of injection of five mL of D5W above multiple insertions, entering 2.5 cm proximal to the left deltoid, triceps, biceps, brachioradialis, pronator teres, and supinator insertions, and injection of one mL of D5W, entering approximately 1.25 cm proximal to the insertion points of the extensor digitorum tendons 2-5.

Video 5 shows manual muscle testing before and after DMIT for multiple muscles with 4/5 weakness in her left upper extremity (LUE) from a CVA eight weeks earlier. All treated muscles showed in-session improvement to 5/5 strength. 

**Video 5:** DMIT for left upper extremity weakness eight weeks after CVA DMIT resulted in all 4/5 LUE muscles improving to 5/5 in minutes at eight weeks (May 26, 2025, through July 21, 2025)

The video from July 21, 2015, shows her within-session improvement in shoulder elevation, elbow extension, and forearm supination and pronation from 4/5 to 5/5, and in hand function. An additional video, performed five years later, is appended, which demonstrates strength maintenance. Further gradual recovery would not be unexpected, given the relatively minor nature of her infarct, but the within-session response of all treated muscles at the point of an eight-week-post-CVA plateau in improvement was particularly remarkable.

## Discussion

Here, we reported five cases utilizing DMIT, none of which required a second treatment. These cases appear to be representative of the responses we have seen with this technique. These cases appear to be representative of the responses we have seen with this technique. These brief case reports suggest the potential for DMIT to result in consistent, prompt, and potentially sustainable muscle recruitment in diverse upper motor neuron conditions. The technique involved subcutaneous injection of D5W near the enthesis of weakened muscles. No ultrasound guidance was used, and the volume varied by muscle size (typically 0.5-5 mL). Injections were delivered into the superficial subcutaneous layer rather than into the muscle belly or nerve trunk. Functional and timeline observation of these patients raises the intriguing possibility that loss of motor control from a variety of proximal lesions or even disuse, when prolonged beyond an undetermined minimum period of time, may result in an apparently stable clinical plateau in muscle recruitment, despite significant and unexpressed interim healing of damage, and that DMIT allows for interim healing effects to be expressed by prompt, significant, and potentially durable additional muscular recruitment. How could such an unexpected change occur after such distant events with an apparently simple and accessible intervention?

The use of dextrose as a primary injectate suggests that mechanistic roots may be shared with therapeutic dextrose injection for chronic musculoskeletal conditions with associated pain and dysfunction [6]. The emphasis of research on the clinical efficacy and neurophysiology of therapeutic dextrose injection has shifted in recent years toward subcutaneous perineural injection of D5W for chronic pain (termed perineural injection treatment [PIT]) [4,5] and ultrasound-guided hydrodissection using D5W for release of nerve entrapment, with favorable clinical trial results for carpal [7,8] and cubital tunnel syndrome [9,10]. Histologic and electromyographic measures evidence improvement in intraneural edema, and conduction study findings [10,11]. In vitro research thus far has suggested that dextrose protects damaged nerve cells by reducing neurogenic inflammation (reducing the levels of inflammatory neuropeptides [12,13]). However, reduction of neurogenic inflammation alone does not explain the speed of response in pain levels after PIT [14], which has been confirmed to occur within minutes in a controlled clinical trial [15] and empirically occurs within seconds. Despite targeting motor recovery rather than pain, the speed of response and the superficial nature of the injections in DMIT mirror PIT’s effects. This overlap justifies revisiting the historical evolution of proposed PIT mechanisms to understand what may underlie DMIT’s impact on motor strength. In so doing, we cannot explain the prompt strength improvement on the basis of improvement in pain, because none of these patients had complaints of pain. Three primary hypotheses have been proposed.

Structural transport hypothesis

Direct effects of dextrose on a bication channel (transient receptor potential vanilloid receptor type 1 [TRPV1], or T-type potassium or calcium channels). The search for a unifying mechanism behind PIT has undergone several evolutions, reflecting both empirical observations and emerging anatomical theories. Initially, PIT practitioners embraced a receptor-based model centered on TRPV1 inhibition [14]. This was grounded in the observation that the fibers being targeted, superficial unmyelinated afferents, were likely C-fibers, and more specifically, the peptidergic subtype known to release substance P and calcitonin gene-related peptide (CGRP). TRPV1 receptors are highly expressed on these peptidergic C-fibers and are involved in the regulation of neuropeptide release. The idea was that dextrose, possibly through an unknown interaction with TRPV1 or related channels, could restore homeostatic balance by modulating SP and CGRP release. However, it was soon recognized that TRPV1 does not have a known dextrose binding site [16], prompting speculation that dextrose might instead modulate T-type calcium or potassium channels that influence TRPV1 activity indirectly. However, molecular evidence has not been found thus far to support direct modulation of other ion channels by dextrose as a rationale for either PIT (or DMIT) effects. 

Energetic hypothesis

Restoration of Energy to Injured Neurons

The energetic hypothesis is that dextrose acts as a metabolic substrate to restore energy to injured neurons via increased ATP production [17]. 

There are two considerations that weaken this hypothesis. First, glucose uptake by neurons is primarily mediated by GLUT1 at the blood-brain barrier and GLUT3 at the neuronal membrane. These transporters have low KM values (~1-5 mM for GLUT3), indicating that they are already functioning near saturation under normal extracellular glucose concentrations (~5 mM) [18]. A subcutaneous injection of 5% dextrose (≈278 mM) certainly creates a supraphysiologic local concentration, but given the saturable nature of GLUT1 and GLUT3, the resultant glucose influx would be nominal. Thus, the capacity for increasing ATP appears to be minimal. Second, increased energy production would take time and would not occur within the 5 to 60 seconds in which PIT effects are empirically observed.

However, glucose uptake by the cell will be dependent on the product of transporter velocity and abundance. As discussed above, it is unlikely that transporter velocity increases because GLUT1 and GLUT3 are saturated under physiological conditions. There is no data to rule out the possibility that the transient rise in glucose triggers an increase in glucose transporter abundance. It remains unclear how a substantial increase in transport abundance would occur within 5 to 60 seconds, but it could contribute to a more prolonged mechanism of action.

Receptor-mediated neuromodulation hypothesis

Han et al. [19] demonstrated that peripheral dextrose injection could induce biochemical and electrophysiologic changes at the spinal cord level within one minute. The study’s findings support a mechanism by which peripheral nerve inputs, likely mediated via C-fibers, could influence central spinal processing rapidly enough to match clinical timelines via a fast, afferent-driven signaling mechanism, potentially serving as the primary driver of both PIT and DMIT clinical effects. Given the rapid expansion of research on neurophysiologic interactions at the cellular and tissue level, any proposal must, of necessity, be preliminary and only partially correct at best. One such proposal for receptor-mediated neuromodulation consists of the following steps. 

Step 1

Subcutaneous dextrose injection stimulates superficial C-fibers [19], which, by anatomic convergence, affect spinal segments supplying motor fibers at the same levels. 

The enthesis, the site where tendon or ligament attaches to bone, is a complex anatomical and sensory region, often innervated by nociceptors and mechanoreceptors, including C-fiber afferents. According to Hilton's Law [20], the same spinal nerves supply the joint, the muscles moving the joint, and the skin overlying it. Extrapolating from this, it’s reasonable to hypothesize that sensory input from subcutaneous injections near the enthesis could influence spinal segments that also govern motor output to the related muscle. 

Step 2

Glucose stimulates activation of the acid-sensing ion channel-1a (ASIC1a) on C-fibers, resulting in transient release of substance P (SP) and related phosphorylation of extracellular signal-regulated kinase (ERK) to phosphorylated ERK (pERK) in dorsal root ganglia (DRG) neurons.

C-fibers, small unmyelinated sensory afferents densely distributed in subcutaneous tissue, have a bidirectional signaling capacity, and their spinal cord connections make them ideal neuromodulatory conduits. Han et al. [19] demonstrated that D5W activates ASIC1a channels on these afferents, triggering SP release and ERK phosphorylation in dorsal root ganglia (DRG) neurons. Blocking ASIC1a or SP abolishes this effect, confirming a causal pathway.

Step 3

SP-associated phosphorylation of ERK to pERK promotes a shift to (activates) an anti-inflammatory (reparative) microglial phenotype. 

Although SP traditionally functions as a pain neurotransmitter, emerging evidence points to context-dependent anti-inflammatory and neuromodulatory roles. Chung et al. [21] showed that intravenous SP in mice with neuropathic pain reduces mechanical allodynia and spinal glial activation in the dorsal horn of the spinal cord via a reduction of pERK and glial fibrillary acidic protein (GFAP) in astrocytes, while upregulating IL-10 and reducing TNF-α expression, indicating a shift to an anti-inflammatory glial phenotype. An SP-associated shift in microglia toward a reparative, M2-like phenotype has also been observed in both ischemic stroke models [22] and glial cell cultures [23]. 

Step 4

Glial disinhibition lifts suppression on motor circuits, restoring function.

In chronic injury states, activated astrocytes and microglia release cytokines that dampen motor output [24,25]. These glial-derived pro-inflammatory signals contribute to sustained inhibition of motor circuits and central sensitization. By reversing this activation state, SP may relieve motor suppression and re-enable spinal motor circuits. This is supported by studies showing that spinal pERK is sequentially elevated in neurons and later in glia during chronic pain [25,26]; SP appears to disrupt this glial ERK maintenance phase.

Other support for receptor-mediated neuromodulation in DMIT includes the following three areas of investigation:

Non-nociceptive modulation by C-fiber subtypes, a conceptual support: Although most models of C-fiber signaling emphasize nociception, recent work shows that certain sub-threshold C-fiber inputs, such as those from low-threshold mechanoreceptors like C-tactile fibers, can modulate spinal circuits in non-nociceptive ways [27]. While direct evidence for their role in motor recovery is lacking, this form of afferent signaling may serve as a broader modulatory input to the central nervous system and can be cautiously interpreted as supportive of the hypothesis that dextrose-induced activation of C-fibers might deliver a restoration or safety signal conducive to motor reactivation.

Dextrose as a non-metabolic physiologic signal: Beyond neural effects, it has been shown that dextrose also acts as a signaling molecule. Lopez-Pajares et al. demonstrated that glucose directly binds and modulates the transcription factor IRF6, promoting its dimerization and enabling epidermal differentiation [28]. This effect occurred independently of metabolism and could be replicated with non-metabolizable glucose analogs. These findings underscore glucose’s role as a non-metabolic “go” signal, capable of directly modulating protein function and cellular behavior, and suggest a plausible parallel mechanism by which dextrose could exert neuromodulatory effects on peripheral nerves and glia, independent of energy metabolism.

Dextrose as an activator of nutrient-sensing pathways: In addition to membrane-bound receptor mechanisms, dextrose may also engage intracellular nutrient-sensing pathways, offering a complementary route through which cells sense high glucose as a permissive signal for repair, growth, and motor reactivation. After injection, compartmentally high dextrose could activate nutrient-sensing pathways or transcriptional programs involved in tissue readiness, plasticity, or cellular reprogramming, thereby contributing to the observed motor improvements. Indeed, dextrose can modulate canonical nutrient-sensing pathways such as adenosine monophosphate (AMP)-activated protein kinase (AMPK) and the mechanistic target of rapamycin (mTOR). AMPK can act as a cellular energy gauge, promoting conservation during low-energy states, while mTOR is activated in high-energy conditions, driving protein synthesis, growth, and regeneration [28,29].

A localized glucose surge, such as that created by subcutaneous injection, may transiently tip this signaling balance toward mTOR activation, favoring anabolic and restorative responses. In this context, dextrose may function not just as a resource but as a signal that the metabolic and immunologic environment is favorable for healing. Dextrose could provide a cue for local glial and neuronal populations to transition from defense to repair, thereby reawakening suppressed circuitry and unlocking latent neuroplasticity.

Limitations of this case report include a lack of timestamping of the videos to objectively confirm a within-session response of these patients. No control treatment was offered, and no formal attempt was made to obtain consecutive cases to confirm that recovery from 4/5 to 5/5 strength was a common response. Cases of 3/5 were not included, as their responses appear to be more variable, not approaching 5/5 power, and less suitable for formal research designs. The authors also realize that although manual muscle testing is the standard used for documenting muscle weakness, it is a qualitative measurement, with potentially significant inter-rater reliability concerns. The difference between a 4/5 and 5/5 muscle is not subtle and can be filmed as shown in the videos included in this report. However, alternative explanations, such as the placebo effect, examiner bias, or voluntary effort increase, cannot be ruled out based on the current data.

The primary strength of these case reports is in the rapid and seemingly durable restoration of motor strength years after an apparent maximal rehabilitation effort with one treatment, which seems inconsistent with a placebo response. The empirically observed consistency of response, speed of response, specificity of injection site, specificity of injectate, and safety of injectate will facilitate practical applications of this technique in animal studies and single- and double-blind human clinical trials. In addition, monitoring of motor unit recruitment in real time, pre- and post-treatment, along with time-stamped concurrent videos, may provide valuable information.

## Conclusions

Dextrose muscle insertion therapy could provide hope for patients with 4/5 motor weakness who have no potent treatment options after plateau, and challenge the long-held belief that motor recovery, or alternatively, peak motor unit recruitment, plateaus permanently after injury. Functionally, this treatment may also offer the opportunity for those with 3/5 motor power to gain sufficient additional power to attain critical ADL goals, such as lifting a leg into a car or up a stair, or holding an arm up for self-feeding or dressing. If confirmed, this treatment could provide practitioners with a way to support the rehabilitation team and patient in attaining higher functional ability after a variety of neurologic insults and will stimulate research on the neurophysiology of recovery after a variety of insults that impact motor function.

## References

[1] Stinear CM, Lang CE, Zeiler S, Byblow WD (2020). Advances and challenges in stroke rehabilitation. Lancet Neurol.

[2] Lim XY, Wong JK, Idris Z (2023). Structured manual muscle testing of the lower limbs. Malays J Med Sci.

[3] Reeves KD, Lyftogt J (2011). Prolotherapy: Regenerative injection therapy. Pain Management.

[4] Yelland MJ, Sweeting KR, Lyftogt JA (2011). Prolotherapy injections and eccentric loading exercises for painful Achilles tendinosis: a randomised trial. Br J Sports Med.

[5] Oh-Park M, Desjardins E, Chater A (2019). Therapeutic Injection of Dextrose: Prolotherapy, Perineural Injection Therapy and Hydrodissection. https://now.aapmr.org/therapeutic-injection-of-dextrose-prolotherapy-perineural-injection-therapy-and-hydrodissection/..

[6] Rabago D, Reeves KD, Doherty MP, Fleck M (2019). Prolotherapy for musculoskeletal pain and disability in low- and middle-income countries. Phys Med Rehabil Clin N Am.

[7] Sveva V, Farì G, Fai A (2024). Safety and efficacy of ultrasound-guided perineural hydrodissection as a minimally invasive treatment in carpal tunnel syndrome: a systematic review. J Pers Med.

[8] Oh MW, Park JI, Shim GY, Kong HH (2025). Comparative efficacy of 5% dextrose and corticosteroid injections in carpal tunnel syndrome: a systematic review and meta-analysis. Arch Phys Med Rehabil.

[9] Chen LC, Ho TY, Shen YP (2020). Perineural dextrose and corticosteroid injections for ulnar neuropathy at the elbow: a randomized double-blind trial. Arch Phys Med Rehabil.

[10] Mansiz-Kaplan B, Nacir B, Pervane-Vural S (2022). Effect of perineural dextrose injection on ulnar neuropathy at the elbow: a randomized, controlled, double-blind study. Arch Phys Med Rehabil.

[11] Wu YT, Ho TY, Chou YC (2017). Six-month efficacy of perineural dextrose for carpal tunnel syndrome: A prospective, randomized, double-blind, controlled trial. Mayo Clin Proc.

[12] Wu YT, Chen YP, Lam KH (2022). Mechanism of glucose water as a neural injection: a perspective on neuroinflammation. Life (Basel).

[13] Cherng JH, Chang SJ, Tsai HD (2023). The potential of glucose treatment to reduce reactive oxygen species production and apoptosis of inflamed neural cells in vitro. Biomedicines.

[14] Lam SK, Reeves KD, Cheng AL (2017). Transition from deep regional blocks toward deep nerve hydrodissection in the upper body and torso: method description and results from a retrospective chart review of the analgesic effect of 5% dextrose water as the primary hydrodissection injectate to enhance safety. Biomed Res Int.

[15] Maniquis-Smigel L, Dean Reeves K, Jeffrey Rosen H (2017). Short term analgesic effects of 5% dextrose epidural injections for chronic low back pain: a randomized controlled trial. Anesth Pain Med.

[16] Caterina M, Schumacher M, Tominaga M (1997). The capsaicin receptor: a heat-activated ion channel in the pain pathway. Nature.

[17] Topol GA, Pestalardo IG, Reeves KD (2022). Dextrose prolotherapy for symptomatic grade IV knee osteoarthritis: a pilot study of early and longer-term analgesia and pain-specific cytokine concentrations. Clin Pract.

[18] Duelli R, Kuschinsky W (2001). Brain glucose transporters: relationship to local energy demand. News Physiol Sci.

[19] Han DS, Lee CH, Shieh YD (2022). A role for substance P and acid-sensing ion channel 1a in prolotherapy with dextrose-mediated analgesia in a mouse model of chronic muscle pain. Pain.

[20] Hébert-Blouin MN, Tubbs RS (2014). Hilton's law revisited. Clin Anat.

[21] Chung E, Yoon TG, Kim S (2017). Intravenous administration of Substance P attenuates mechanical allodynia following nerve injury by regulating neuropathic pain-related factors. Biomol Ther (Seoul).

[22] Ahn W, Chi G, Kim S (2023). Substance P reduces infarct size and mortality after ischemic stroke, possibly through the M2 polarization of microglia/macrophages and neuroprotection in the ischemic rat brain. Cell Mol Neurobiol.

[23] Kim S, Son Y (2021). Astrocytes stimulate microglial proliferation and M2 polarization in vitro through crosstalk between astrocytes and microglia. Int J Mol Sci.

[24] Milligan ED, Watkins LR (2009). Pathological and protective roles of glia in chronic pain. Nat Rev Neurosci.

[25] Xiong Y, Chen J, Li Y (2023). Microglia and astrocytes underlie neuroinflammation and synaptic susceptibility in autism spectrum disorder. Front Neurosci.

[26] Zhuang ZY, Gerner P, Woolf CJ, Ji RR (2005). ERK is sequentially activated in neurons, microglia, and astrocytes by spinal nerve ligation and contributes to mechanical allodynia in this neuropathic pain model. Pain.

[27] Larsson M, Nagi SS (2022). Role of C-tactile fibers in pain modulation: animal and human perspectives. Cur Opi Beh Sci.

[28] Lopez-Pajares V, Bhaduri A, Zhao Y (2025). Glucose modulates IRF6 transcription factor dimerization to enable epidermal differentiation. Cell Stem Cell.

[29] Leprivier G, Rotblat B (2020). How does mTOR sense glucose starvation? AMPK is the usual suspect. Cell Death Discov.

